# Dietary Quality during Pregnancy and Congenital Heart Defects

**DOI:** 10.3390/nu14173654

**Published:** 2022-09-04

**Authors:** Jiaomei Yang, Qianqian Chang, Shaonong Dang, Xin Liu, Lingxia Zeng, Hong Yan

**Affiliations:** 1 Department of Epidemiology and Health Statistics, School of Public Health, Xi’an Jiaotong University Health Science Center, Xi’an 710061, China; 2 Nutrition and Food Safety Engineering Research Center of Shaanxi Province, Xi’an 710061, China; 3 Key Laboratory of Environment and Genes Related to Diseases, Xi’an Jiaotong University, Ministry of Education, Xi’an 710061, China

**Keywords:** dietary quality, congenital heart defects, pregnancy, Global Diet Quality Score, Mediterranean Diet Score

## Abstract

Limited studies on maternal dietary quality indices and congenital heart defects (CHD) are available. This study aimed to explore the relationship between dietary quality in pregnancy and CHD among the Chinese population. A case-control study was performed in Northwest China, and 474 cases and 948 controls were included. Eligible women waiting for delivery were interviewed to recall diets and other information during pregnancy. Dietary quality was assessed by the Global Diet Quality Score (GDQS) and Mediterranean Diet Score (MDS). Logistic regression models were adopted to evaluate the associations of dietary quality scores with CHD. Pregnant women with higher scores of GDQS and MDS were at a lower risk of fetal CHD, and the adjusted ORs comparing the extreme quartiles were 0.26 (95%CI: 0.16–0.42; *P*_trend_ < 0.001) and 0.53 (95%CI: 0.34–0.83; *P*_trend_ = 0.007), respectively. The inverse associations of GDQS and MDS with CHD appeared to be stronger among women with lower education levels or in rural areas. Maternal GDQS and MDS had good predictive values for fetal CHD, with the areas under the receiver operating characteristic curves close to 0.8. Efforts to improve maternal dietary quality need to be strengthened to decrease the prevalence of CHD among the Chinese population.

## 1. Introduction

Congenital heart defects (CHD) refer to the structural abnormalities of the heart and/or vessels at birth. As the most common congenital anomaly worldwide, the CHD birth prevalence is estimated to be 9.41‰, with millions of newborns diagnosed with CHD each year [1]. In China, the estimated CHD prevalence at live birth is about 9.00‰, with more than 0.15 million incident cases yearly [2]. CHD accounted for over 0.26 million deaths worldwide in 2017, and remained the leading cause of infant morbidity and mortality from congenital abnormalities [3]. Nowadays nearly 20 million people live with CHD globally, causing great burdens on the family and society [3]. Although some environmental and genetic factors have been generally accepted as the risk factors for CHDs, the etiology of CHD remains to be largely unclear [4,5].

Maternal diet in pregnancy, as an important modifiable factor, has been the focus of interventions to improve birth outcomes because of the low cost and low risk. Existing evidence suggests that maternal low intakes of some nutrients including iron, zinc, selenium, folate, and niacin increase the risk of CHD [6,7,8,9,10], while maternal high intake of vitamin E increase the risk of CHD [11]. Previous researches also suggest that mothers with excessive intake of barbecued foods, smoked foods, fried foods, and pickled vegetables are at higher risks of fetal CHD and ventricular septal defects (VSD), while mothers with regular intake of fresh fruits, dairy, and fish and shrimp are at lower risks of fetal CHD and VSD [12,13,14]. However, most studies on the association between nutrition and CHD to date focus on individual nutrients or foods, which does not fully capture the complex interactions among nutrients and foods. Despite the emphasis of recent dietary recommendations on healthy dietary patterns, limited studies have been published on optimal dietary patterns in pregnancy to reduce the risk of CHD [15,16,17,18], especially for the use of dietary quality indices [18]. Dietary quality indices have great potential for use among people because of their easier collection and interpretation, especially in low- and middle-income countries [19,20]. However, the associations between maternal dietary quality indices and CHD have not been investigated in the population except one in America [18].

The Global Diet Quality Score (GDQS) is a novel food group-based dietary score according to the data from 14 countries [21]. The GDQS has the ability to capture nutrient adequacy and diet-related non-communicable disease, and is a promising candidate for global monitoring platforms [21]. The Mediterranean Diet Score (MDS) is developed on the basis of the Mediterranean diet, which is high in fruits, vegetables, whole grains, legumes, fish, and nuts, high in olive oil but low in saturated lipids, low to moderate in dairy, and limited in red meat and wine [22]. Previous studies have shown that the Mediterranean diet in pregnancy benefits maternal and offspring health [19]. To our knowledge, the GDQS has not been assessed for the associations with pregnancy outcomes including CHD. For the MDS, only one study in America has used it to examine the association with CHD [18]. It remains unknown whether maternal MDS during pregnancy could be used to evaluate the association with CHD in Asian countries, where the dietary habits are distinct from those in western countries. The prevalence of CHD (the mild lesions in particular) is higher in Asia than that in other regions, and the increase rate of CHD prevalence (atrial septal defects (ASD) in particular) in Asia is also greater than in other regions [1], which may partly come from the difference in dietary habits. Some studies have described maternal predictors for CHD [23,24,25,26], providing a reference for the early prediction of CHD. However, the prediction values for dietary quality indices on CHD have not been explored.

The current study in Northwest China aimed to explore the associations of maternal dietary quality during pregnancy assessed by GDQS and MDS with the risk of CHD, and evaluate the prediction values for dietary quality indices on CHD.

## 2. Materials and Methods

### 2.1. Study Design and Participants

We performed a case-control study in six comprehensive hospitals from August 2014 to August 2016 in Xi’an City, Northwest China. These six hospitals were selected according to their qualification to perform the diagnoses of birth defects and their willingness to cooperate. The detailed fetal echocardiography during the 20th–24th month of gestation was in the routine prenatal screening program in the six hospitals, and used as the prenatal diagnosis of CHD. The study design has been published in detail previously [8,15]. Briefly, we recruited participants among the pregnant women waiting for delivery in the cooperated hospitals. Pregnant women whose fetuses had isolated CHD and no genetic abnormalities were included in the case group, and pregnant women whose fetuses had no diagnosed congenital anomalies were included in the control group. Mothers with multiple gestations or diabetes were excluded because of potentially different etiologies. Qualified specialists in the ultrasound, obstetrics, and pediatrics departments strictly enforced the diagnostic standard criteria to finish the diagnoses in cases and controls. We further conducted a telephone follow-up within one year after birth to confirm the diagnosis. All the CHD diagnoses were ascertained by echocardiography and/or cardiac catheterization and/or surgery. We randomly selected controls each month in each hospital, and the ratio of the number of controls to cases included in the same month in the same hospital was 2:1. To detect a significant (*p* < 0.05) OR of 0.75 between groups of good and poor dietary quality with a statistical power of 80%, 443 cases, and 886 controls would be required. Finally, 474 cases and 948 controls with completed questionnaires were included, meeting the requirements of the sample size.

All participants gave written informed consent. The ethics committee of Xi’an Jiaotong University Health Science Center approved this study (No. 2012008).

### 2.2. Dietary Quality Evaluation

We used a 111-item semi-quantitative food frequency questionnaire (FFQ) to collect maternal diets throughout the entire pregnancy when women were waiting for delivery in the hospital. The median time from the end of the interview to delivery was two days among the cases and controls. Maternal dietary habits tend to be stable across pregnancy [27]; thus, maternal diets during the entire pregnancy are comparable with those in the 3rd–8th week of gestation, the critical period of cardiac development [6,8,15,28]. The FFQ was established according to a validated FFQ designed for pregnant women in Northwest China [29]. Women recalled consumption frequency based on eight predefined categories ranging from never to two or more times per day and reported portion sizes with the help of food portion images [30,31]. The nutrient contents of foods were derived from the Chinese Food Composition Tables [32,33].

The GDQS and MDS were used to assess dietary quality because these priori-defined indices were previously validated and reflected common recommended dietary guidelines. The GDQS consists of 16 healthy food groups (citrus fruits, deep orange fruits, other fruits, dark green leafy vegetables, cruciferous vegetables, deep orange vegetables, other vegetables, legumes, deep orange tubers, nuts and seeds, whole grains, liquid oils, fish and shellfish, poultry and game meat, low-fat dairy, and eggs), 7 unhealthy food groups (processed meat, refined grains, and baked goods, sweets and ice cream, sugar-sweetened beverages, juice, white roots and tubers, and purchased deep fried foods), and 2 food groups regarded as unhealthy in excessive amounts (high-fat dairy, and red meat) [21]. The daily intake of each food group was classified into 3 or 4 categories. For 16 healthy food groups, points from 0 to 4 were given to each intake category, with higher intake receiving more points. For the other 9 food groups, points from 0 to 2 were given according to the intake categories. The GDQS was a sum of all 25 food group scores, with a range of 0 to 49 [21]. The higher the GDQS was, the better the diet quality was. A previously modified version of MDS for pregnant women was used in this study [34], in which 8 components were positively scored (fruits, vegetables, legumes, whole grains, fish, dairy, nuts, monounsaturated-to-saturated fat ratio), and 1 component was negatively scored (red and processed meat). Zero or one points were assigned according to the median intake for each component. The MDS was summed for each component score, and the range was 0 to 9, with higher scores showing better adherence to the Mediterranean diet. Alcohol consumption was excluded from the original MDS because alcohol intake was not recommended during pregnancy and our participants rarely drank alcohol during pregnancy.

### 2.3. Covariates

We collected the general information about pregnancy face-to-face by a standard questionnaire. The study covariates included (1) socio-demographic characteristics: maternal age (<30 years/≥30 years), work (in employment/without employment), education (junior high school or below/senior high school or above), residence (urban/rural), and parity (0/≥1); (2) maternal health-related factors in early pregnancy: passive smoking (no/yes), anemia (no/yes), medication use (no/yes), and folate/iron supplements use (yes/no). Women with no paid employment outside their homes were regarded as without employment. People exposed to others’ tobacco smoke for ≥15 min/d was defined as exposure to passive smoking. Women with hemoglobin concentration <110 g/L in early pregnancy were diagnosed with anemia.

### 2.4. Statistical Analysis

Univariate comparisons between groups were tested by the χ^2^ test for categorical variables, and by the Mann-Whitney U test for continuous variables because of the non-normal distributions observed by the Shapiro–Wilk test. Considering the clustering in the design through hospitals, we used mixed logistic regression models to estimate ORs (95%CIs) for total CHDs and CHDs subtypes associated with GDQS and MDS. The GDQS and MDS were divided into four groups according to quartiles of the control distribution. The confounding variables were adjusted in the models if they were reported to be risk factors for CHD [4,5] and changed the estimated by more than 10% [35], which finally included maternal age, work, education, residence, parity, and maternal passive smoking, anemia, medication use, and folate/iron supplement use in early pregnancy. Total energy intake during pregnancy was adjusted in the models to indirectly reflect maternal BMI status that was not collected in the survey. *p* for trend was calculated by including quartile-specific median value in the model. The risk of CHD associated with per 1 higher score of GDQS and MDS was assessed by mixed logistic regression models. Subgroup analyses were performed by baseline characteristics including maternal age, work, education, residence, parity, and maternal passive smoking, anemia, medication use, and folate/iron supplement use in early pregnancy. The interaction between GDQS or MDS and each of the subgroup factors was tested by the likelihood ratio test comparing regression models with and without an interaction term. The receiver operating characteristic curves (ROC) were constructed to determine the optimal cut-off values of GDQS and MDS in pregnancy for CHD with the maximum Youden index (sensitivity + specificity − 1). The areas under the ROC (AUCs) showed the accuracy of GDQS and MDS as predictive markers for CHD. When the AUC > 0.5, the closer the AUC was to 1, the better the predictive power of the model was, as follows: AUC > 0.9, very good; AUC > 0.8, good; and AUC > 0.7, useful [36].

We used the Stata software (version 15.0; StataCorp, College Station, TX, USA) to conduct the statistical analyses. We set the statistical significance at 0.05 with two-sided.

## 3. Results

### 3.1. Baseline Characteristics of the Study Population

Among the 474 CHD babies, 46.8% were diagnosed with VSD, and 46.0% with ASD, followed by atrioventricular septal defects, patent ductus arteriosus, and tetralogy of fallot (Table S1). Women in the cases were less likely to be in employment, have higher education levels, live in an urban area, and be nulliparity when compared with those in the controls (Table 1). Passive smoking, anemia, and medication use in early pregnancy were more common in the cases than in the controls, while folate/iron supplements use in early pregnancy was less common in the cases than in the controls. The proportion of babies with birth weight lower than 2500 g was higher in the cases than in the controls, while no difference in gestational age was found between the two groups. Pregnant women in the cases had lower intakes of energy and most nutrients except carbohydrate in comparison with those in the controls. Women in the cases had significantly lower GDQS and MDS in pregnancy than the controls (both *p* < 0.001).

### 3.2. The Distribution of Food Components in GDQS and MDS among Cases and Controls

**Figure 1:**
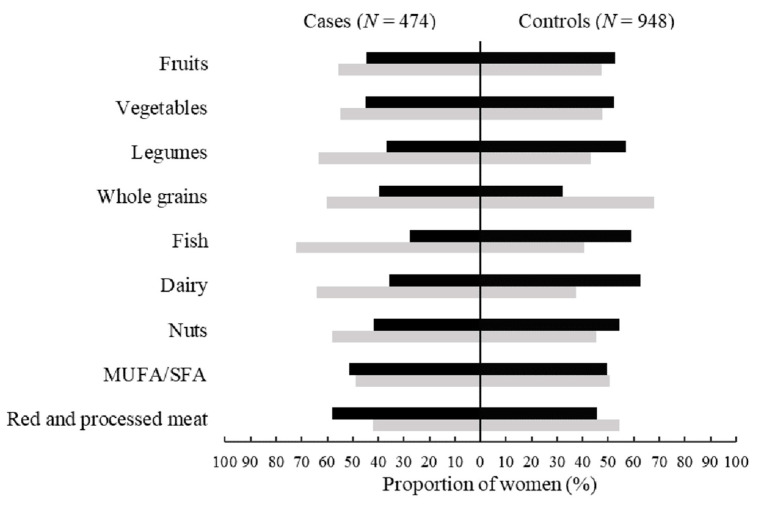
The proportion of women consuming food components in the Mediterranean Diet Score during pregnancy by the median intake levels among cases and controls. MUFA/SFA, monounsaturated-to-saturated fat ratio. Dark shaded bars indicate maternal consumption above the median levels, and light shaded bars indicate maternal consumption equal to or below the median levels. Statistically significant differences were found for the groups of fruits, vegetables, legumes, fish, dairy, nuts, and red and processed meat between cases and controls by the χ^2^ test (all *p* < 0.05).

The GDQS scores for all 16 healthy food groups except dark green leafy vegetables, whole grains, and low-fat dairy were significantly lower in the cases than in the controls (all *p* < 0.05) (Table 2). The GDQS scores for juice, purchased deep-fried foods, high-fat dairy, and red meat were also significantly lower in the cases than the controls (all *p* < 0.05), while the GDQS scores for the other five unhealthy food groups were not significantly different between groups (Table 2). The proportions of women consuming fruits, vegetables, legumes, fish, dairy, and nuts above the median intake levels during pregnancy were significantly lower in the cases than in the controls, while the proportion of women consuming red and processed meat above the median intake level during pregnancy was significantly higher in the cases than the controls (Figure 1).

Daily nutrient intakes during pregnancy by GDQS and MDS categories among cases and controls are shown in Table S2 and Table S3, respectively. In both cases and controls, daily intakes of energy, macronutrients, and micronutrients were increased with higher scores of GDQS and MDS (all *p* < 0.001).

### 3.3. Associations of Maternal GDQS and MDS during Pregnancy with CHD

The risk of total CHD was reduced with the increasing quartiles of GDQS and MDS, and the tests for trend were statistically significant (both *p* for trend <0.05) (Table 3). The fully adjusted ORs comparing the highest with the lowest quartiles of the GDQS and MDS were 0.26 (95%CI: 0.16–0.42) and 0.53 (95%CI: 0.34–0.83), respectively. The risk of total CHD was reduced by 12% (OR: 0.88, 95%CI: 0.85–0.91) and 12% (OR: 0.88, 95%CI: 0.80–0.95) for per 1 higher score of GDQS and MDS, respectively. The fully adjusted models also showed inverse associations of GDQS and MDS with the risks of VSD and ASD (all *p* for trend <0.05) (Table 3). Per 1 higher score of GDQS and MDS was associated with 12% (OR: 0.88, 95%CI: 0.84–0.92) and 11% (OR: 0.89, 95%CI: 0.81–0.98) lower risk of VSD, respectively. Per 1 higher score of GDQS and MDS was associated with 13% (OR: 0.87, 95%CI: 0.84–0.91) and 10% (OR: 0.90, 95%CI: 0.83–0.98) lower risk of ASD, respectively.

Subgroup analyses showed that the associations of maternal GDQS and MDS during pregnancy with total CHD did not change by maternal age, work, parity, or maternal health-related factors in early pregnancy (passive smoking, anemia, medication use, or folate/iron supplements use) (Figures S1 and S2). However, the inverse association between maternal GDQS and the risk of total CHD appeared to be stronger among women with lower education levels and in rural areas, and the tests for interaction were significant (both *p* < 0.05) (Figure S1). The inverse association between maternal MDS and the risk of total CHD appeared to be stronger among women in rural areas, and the test for interaction was significant (*p* = 0.004) (Figure S2).

### 3.4. The Prediction Values for Maternal GDQS and MDS during Pregnancy on CHD

**Figure 2:**
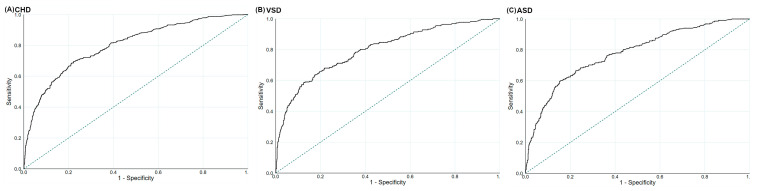
The ROC for the Global Diet Quality Score during pregnancy in the prediction of (**A**) total congenital heart defects, (**B**) ventricular septal defects, and (**C**) atrial septal defects. ASD, atrial septal defects; CHD, congenital heart defects; ROC, receiver operating characteristic curves; VSD, ventricular septal defects. The dotted line refers to the reference line, resulting from random selection.

**Figure 3:**
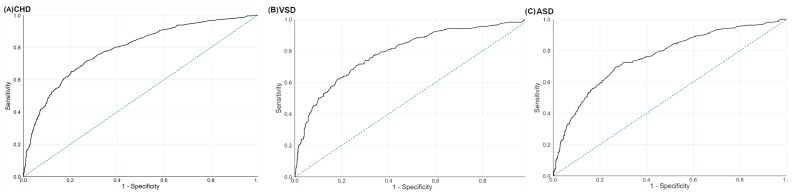
The ROC for the Mediterranean Diet Score during pregnancy in the prediction of (**A**) total congenital heart defects, (**B**) ventricular septal defects, and (**C**) atrial septal defects. ASD, atrial septal defects; CHD, congenital heart defects; ROC, receiver operating characteristic curves; VSD, ventricular septal defects. The dotted line refers to the reference line, resulting from random selection.

The ROC suggested that the performances of maternal GDQS during pregnancy were useful in predicting total CHD, VSD, and ASD, with the AUCs being 0.80 (95%CI: 0.78–0.83), 0.80 (95%CI: 0.76–0.83), and 0.78 (95%CI: 0.74–0.81), respectively (Figure 2). The optimal GDQS cut-off scores were 30 for total CHD (sensitivity: 69.6%; specificity: 77.7%), 28 for VSD (sensitivity: 63.5%; specificity: 82.7%), and 30 for ASD (sensitivity: 60.6%; specificity: 84.8%), respectively. The ROC also suggested that the performances of maternal MDS during pregnancy were useful in predicting total CHD, VSD, and ASD, with the AUCs being 0.79 (95%CI: 0.76–0.81), 0.79 (95%CI: 0.76–0.82), and 0.77 (95%CI: 0.74–0.80), respectively (Figure 3). The optimal MDS cut-off scores were 8 for total CHD (sensitivity: 65.2%; specificity: 79.5%), 7 for VSD (sensitivity: 70.7%; specificity: 73.7%), and 7 for ASD (sensitivity: 69.3%; specificity: 73.5%), respectively.

## 4. Discussion

This case-control study suggested that pregnant women with better dietary quality, defined by higher scores of GDQS and MDS, had a reduced risk of having fetuses with total CHD and subtypes. The inverse associations of maternal GDQS and MDS during pregnancy with CHD appeared to be stronger among women with lower education levels or in rural areas. This case-control study also suggested that maternal GDQS and MDS during pregnancy had good predictive values for total CHD and the subtypes in fetuses, with the AUCs close to 0.8.

To date, there was only one study investigating the relationship between maternal dietary quality and CHD [18]. This previous study conducted in America found that better dietary quality, assessed by MDS and Dietary Quality Index for Pregnancy, reduced the risk of CHD, which was consistent with the results in the current study. Some studies evaluated priori dietary quality indices in association with birth defects [37,38,39], but not CHD. These studies reported significant inverse associations of maternal MDS with the risks of some birth defects (orofacial clefts, neural tube defects, and gastroschisis) [37,38], but not hypospadias [40]. There were three studies examining the risk of CHD in relation to dietary patterns identified by posterior statistical analyses [15,16,17], rather than the priori indices. A study from America reported that a prudent dietary pattern high in reduced-fat milk, yogurt, fortified cereal, whole-wheat bread, and fish, and low in vegetables and fruits reduced CHD risk [16]. The study in the Netherlands observed that the one-carbon-rich dietary pattern, which was high in fish and seafood, lowered the CHD risk [17]. The study in Shaanxi China found that the prudent dietary pattern, which was high in white meats, red meats, vegetables, legumes, dairy, and snacks, and the dairy and egg pattern, which was high in dairy, nuts, and eggs, and low in beverages, decreased the CHD risk [16]. Although these three studies have identified some posterior dietary patterns associated with CHD, it could hardly be applied in other populations because of the subjective. Our study focused on the priori dietary quality indices, to be more easily reproducible and comparable to prior related studies. The magnitude of the risk for CHD associated with maternal MDS in our study was approximately similar to that observed for CHD in the previous study, with the lowest OR comparing the extreme quartiles being 0.53 (95%CI: 0.34–0.83) and 0.43 (95%CI: 0.25–0.75), respectively [18]. The strength of the inverse association between dietary quality and CHD risk seemed to be stronger for GDQS than MDS in the current study, similar to the stronger association for Dietary Quality Index for Pregnancy than MDS with CHD and other birth defects in the previous studies [18,37]. Since there has been no study investigating health outcomes in relation to maternal GDQS in pregnancy, it is hard for us to compare the results about GDQS in our study with other studies. More studies are needed to explore the relationships between maternal GDQS and health outcomes including CHD and to validate the prediction of maternal GDQS on health outcomes.

Maternal diet is critical to fetal growth and development. Suboptimal diets in pregnancy can cause adverse pregnancy outcomes including congenital abnormalities [28]. Maternal low intakes of zinc, selenium, iron, niacin, and folate have been reported to reduce the risk of CHD [6,7,8,11]. Maternal diets rich in these nutrients may benefit the development of the cardiovascular system in fetuses. Women with higher scores of GDQS and MDS during pregnancy had higher intakes of these micronutrients in the current study, which may partly explain the protective effect of better dietary quality on CHD. Pregnant women with better dietary quality may have more opportunities to obtain nutrition knowledge and pay more attention to healthy diets, and thus have better nutritional status [15]. In addition, maternal adherence to better dietary quality was reported to decrease maternal oxidative DNA damage and lipid oxidation [39], which may further benefit the normal development of the fetal cardiovascular system [41]. Maternal dietary quality is also likely to be a mediator of metabolic diseases including gestational diabetes mellitus and hypertension in pregnancy, which further influences the fetal cardiovascular system [42]. However, we could not further conduct the mediation analyses because women with diabetes have been excluded and there were only two women with gestational hypertension.

The current study provides valuable evidence for the relationship between maternal dietary quality in pregnancy and the risk of CHD. However, we should acknowledge some limitations. First, due to the limited sample size, the associations of dietary quality with other CHD subtypes could not be separately investigated. Further studies with large sample sizes are needed to explore the association with other CHD subtypes. Second, selection bias cannot be excluded because pregnant women having CHD fetuses tend to choose comprehensive hospitals for delivery. Selection bias may also come from the fact that this study did not recruit CHD fetuses that did not survive. Third, recall bias cannot be excluded because maternal information during pregnancy was retrospectively reported by mothers waiting for delivery. However, previous studies have indicated that diets and events in pregnancy can be recalled well after years [43,44]. To reduce this bias, standard questionnaires and supporting materials such as food portion images and calendars were used to collect information to help participants recall accurately in the survey. Fourth, exposure misclassification may cause because we collected dietary information during the entire pregnancy, rather than in the critical period of heart development in the 3rd–8th week of gestation. However, previous studies have shown that maternal dietary habits tend to be stable throughout pregnancy [27]. Finally, the possibility of residual confounding from unobserved and unknown factors cannot be excluded. For example, we did not collect information on gestational weight gain or BMI, which was reported to be associated with fetal CHD [45]. In fact, the real causal relationship between dietary quality during pregnancy and CHD cannot be revealed in the case-control study. Further intervention studies are needed to examine the influence of maternal dietary quality on fetal CHD.

## 5. Conclusions

In summary, this case-control study suggested that maternal GDQS and MDS, indicators of dietary quality, were negatively associated with the CHD risk. Moreover, maternal GDQS and MDS during pregnancy had good predictive values for CHD in offspring. These results implied the importance of improving dietary quality in pregnancy to decrease the prevalence of CHD in Northwest China. Further research is warranted to assess the validity of these scoring systems as predictive tools for CHD in other populations.

## Figures and Tables

**Table 1:** Baseline characteristics of the study population.

	Case (*N* = 474)	Control (*N* = 948)	*p* ^1^
Sociodemographic characteristics, %			
Maternal age < 30 years	66.5	65.8	0.812
Maternal work, in employment	50.6	78.7	<0.001
Maternal education, senior high school or above	58.9	80.7	<0.001
Urban residence	66.0	71.6	0.030
Nulliparity	57.8	80.3	<0.001
Maternal health-related factors in early pregnancy, %			
Passive smoking	33.5	9.3	<0.001
Anemia	16.9	10.9	0.001
Medication use	41.6	30.4	<0.001
Folate/iron supplements use	76.6	89.2	<0.001
Birth weight < 2500 g, %	9.7	5.3	0.003
Gestational age < 37 weeks, %	6.1	5.1	0.407
Daily nutrients intakes during pregnancy, median (25th percentile, 75th percentile)			
Total energy, kcal	1753.2 (1452.4, 2086.1)	1907.1 (1563.3, 2415.9)	<0.001
Protein, g	44.5 (32.0, 60.5)	56.9 (40.9, 78.9)	<0.001
Fat, g	30.9 (19.0, 47.8)	41.7 (29.0, 59.5)	<0.001
Monounsaturated fatty acid, g	6.9 (3.9, 12.2)	9.7 (6.6, 14.4)	<0.001
Saturated fatty acid, g	13.1 (8.1, 19.3)	17.4 (12.6, 25.0)	<0.001
Carbohydrate, g	185.6 (142.0, 237.7)	190.9 (142.5, 269.8)	0.057
Iron, mg	17.5 (12.6, 23.3)	20.4 (14.3, 28.9)	<0.001
Zinc, mg	4.7 (3.1, 6.8)	6.4 (4.6, 9.1)	<0.001
Selenium, μg	22.7 (15.0, 32.5)	30.8 (21.9, 43.7)	<0.001
Calcium, mg	457.4 (315.8, 643.8)	500.9 (359.8, 707.0)	<0.001
Niacin, mg	9.6 (7.3, 13.2)	12.5 (9.0, 17.3)	<0.001
Folate, μg	195.1 (161.3, 242.9)	220.2 (181.5, 270.5)	<0.001
Vitamin C, mg	63.5 (42.1, 107.0)	77.0 (51.8, 123.2)	<0.001
Dietary quality scores, median (25th percentile, 75th percentile)			
GDQS	27.5 (23.7, 31.0)	31.0 (27.3, 34.3)	<0.001
MDS	4.0 (2.0, 5.0)	5.0 (3.0, 6.0)	<0.001

GDQS, Global Diet Quality Score; MDS, Mediterranean Diet Score; ^1^ Categorical variables are compared between groups by the χ^2^ test, and continuous variables are compared between groups by the Mann–Whitney U test.

**Table 2:** The distribution of food components in the Global Diet Quality Score among cases and controls.

	Scoring Ranges ^1^ (Cutoffs, g/d) Low/Middle/High	GDQS Subscores	Case (*N* = 474)				Control (*N* = 948)				*p* ^2^
			Low, %	Middle, %	High, %	Score ^2^	Low, %	Middle, %	High, %	Score ^2^	
Citrus fruits	<24/24–69/>69	0, 1, 2	69.6	17.9	12.4	0.0 (0.0, 1.0)	62.6	26.1	11.4	0.0 (0.0, 1.0)	0.032
Deep orange fruits	<25/25–123/>123	0, 1, 2	96.8	3.2	0	0.0 (0.0, 0.0)	93.4	6.6	0	0.0 (0.0, 0.0)	0.007
Other fruits	<27/27–107/>107	0, 1, 2	0.8	13.7	85.4	2.0 (2.0, 2.0)	0.3	4.4	95.3	2.0 (2.0, 2.0)	<0.001
Dark green leafy vegetables	<13/13–37/>37	0, 2, 4	6.8	42.4	50.8	4.0 (2.0, 4.0)	6.5	39.7	53.8	4.0 (2.0, 4.0)	0.321
Cruciferous vegetables	<13/13–36/>36	0, 0.25, 0.5	7.0	43.7	49.4	0.25 (0.25, 0.5)	2.7	37.8	59.5	0.5 (0.25, 0.5)	<0.001
Deep orange vegetables	<9/9–45/>45	0, 0.25, 0.5	33.3	51.1	15.6	0.25 (0.0, 0.25)	20.8	63.1	16.1	0.25 (0.25, 0.25)	<0.001
Other vegetables	<23/23–114/>114	0, 0.25, 0.5	0.4	25.9	73.6	0.5 (0.25, 0.5)	0.3	15.2	84.5	0.5 (0.5, 0.5)	<0.001
Legumes	<9/9–42/>42	0, 2, 4	14.1	40.1	45.8	2.0 (2.0, 4.0)	1.9	27.8	70.3	4.0 (2.0, 4.0)	<0.001
Deep orange tubers	<12/12–63/>63	0, 0.25, 0.5	74.9	21.7	3.4	0.0 (0.0, 0.25)	57.4	40.4	2.2	0.0 (0.0, 0.25)	<0.001
Nuts and seeds	<7/7–13/>13	0, 2, 4	48.9	14.1	36.9	2.0 (0.0, 4.0)	35.2	18.0	46.7	2.0 (0.0, 4.0)	<0.001
Whole grains	<8/8–13/>13	0, 1, 2	29.1	11.0	59.9	2.0 (0.0, 2.0)	32.0	15.5	52.5	2.0 (0.0, 2.0)	0.082
Liquid oils	<2/2–7.5/>7.5	0, 1, 2	0	33.1	66.9	2.0 (1.0, 2.0)	0	27.4	72.6	2.0 (1.0, 2.0)	0.026
Fish and shellfish	<14/14–71/>71	0, 1, 2	69.0	26.4	4.6	0.0 (0.0, 1.0)	38.1	49.3	12.7	1.0 (0.0, 1.0)	<0.001
Poultry and game meat	<16/16–44/>44	0, 1, 2	93.2	5.1	1.7	0.0 (0.0, 0.0)	81.6	16.6	1.8	0.0 (0.0, 0.0)	<0.001
Low-fat dairy	<33/33–132/>132	0, 1, 2	100.0	0	0	0.0 (0.0, 0.0)	100.0	0	0	0.0 (0.0, 0.0)	1.000
Eggs	<6/6–32/>32	0, 1, 2	27.2	35.0	37.8	1.0 (0.0, 2.0)	8.4	34.9	56.6	2.0 (1.0, 2.0)	<0.001
Processed meat	<9/9–30/>30	2, 1, 0	74.9	19.6	5.5	2.0 (1.0, 2.0)	78.7	18.7	2.6	2.0 (2.0, 2.0)	0.069
Refined grains and baked goods	<7/7–33/>33	2, 1, 0	0	0.6	99.4	0.0 (0.0, 0.0)	0.1	0.2	99.7	0.0 (0.0, 0.0)	0.387
Sweets and ice cream	<13/13–37/>37	2, 1, 0	93.9	3.8	2.3	2.0 (2.0, 2.0)	94.2	4.2	1.6	2.0 (2.0, 2.0)	0.790
Sugar-sweetened beverages	<57/57–180/>180	2, 1, 0	98.3	0.8	0.8	2.0 (2.0, 2.0)	99.4	0.6	0	2.0 (2.0, 2.0)	0.056
Juice	<36/36–144/>144	2, 1, 0	94.9	3.2	1.9	2.0 (2.0, 2.0)	98.5	1.4	0.1	2.0 (2.0, 2.0)	<0.001
White roots and tubers	<27/27–107/>107	2, 1, 0	55.9	39.9	4.2	2.0 (1.0, 2.0)	53.9	44.7	1.4	2.0 (1.0, 2.0)	0.814
Purchased deep-fried foods	<9/9–45/>45	2, 1, 0	87.3	10.1	2.5	2.0 (2.0, 2.0)	90.6	9.1	0.3	2.0 (2.0, 2.0)	0.044
High-fat dairy	<35/35–142/142–734/>734	0, 1, 2, 0	46.0	26.2/27.6	0.2	1.0 (0.0, 2.0)	10.4	41.4/47.9	0.3	1.0 (1.0, 2.0)	<0.001
Red meat	<9/9–46/>46	0, 1, 0	27.4	41.6	31.0	0.0 (0.0, 1.0)	7.2	51.6	41.2	1.0 (0.0, 1.0)	<0.001

GDQS, Global Diet Quality Score. ^1^ Scoring ranges: the 3 categories here are low, middle, and high separated by a solidus; for high-fat dairy, 4 categories were classified: low, lower middle, high middle, and high (from left to right). ^2^ Values are present as median (25th percentile, 75th percentile), and compared between groups by Mann–Whitney U test.

**Table 3:** Associations of maternal GDQS and MDS during pregnancy with congenital heart defects.

	Total Congenital Heart Defects (*N*_cases_ = 474)			Ventricular Septal Defects (*N*_cases_ = 222)		Atrial Septal Defects (*N*_cases_ = 218)	
	*N*_cases_/*N*_controls_	Unadjusted OR (95%CI)	Adjusted OR (95%CI) ^1^	Unadjusted OR (95%CI)	Adjusted OR (95%CI) ^1^	Unadjusted OR (95%CI)	Adjusted OR (95%CI) ^1^
**GDQS**							
Quartile 1	218/218	1	1	1	1	1	1
Quartile 2	131/241	0.54 (0.41, 0.72)	0.59 (0.42, 0.82)	0.51 (0.35, 0.74)	0.51 (0.32, 0.79)	0.57 (0.39, 0.83)	0.51 (0.34, 0.79)
Quartile 3	75/237	0.32 (0.23, 0.44)	0.36 (0.24, 0.53)	0.35 (0.23, 0.52)	0.38 (0.23, 0.63)	0.39 (0.26, 0.59)	0.35 (0.21, 0.59)
Quartile 4	50/252	0.20 (0.14, 0.28)	0.26 (0.16, 0.42)	0.18 (0.11, 0.29)	0.23 (0.12, 0.44)	0.21 (0.13, 0.34)	0.21 (0.11, 0.41)
*p* for trend	474/948	<0.001	<0.001	<0.001	<0.001	<0.001	<0.001
Per 1 higher score	474/948	0.87 (0.85, 0.89)	0.88 (0.85, 0.91)	0.87 (0.84, 0.90)	0.88 (0.84, 0.92)	0.88 (0.85, 0.91)	0.87 (0.84, 0.91)
**MDS**							
Quartile 1	142/164	1	1	1	1	1	1
Quartile 2	159/271	0.67 (0.50, 0.91)	0.76 (0.54, 1.08)	0.68 (0.46, 1.01)	0.87 (0.55, 1.38)	0.80 (0.53, 1.19)	0.83 (0.53, 1.31)
Quartile 3	75/162	0.53 (0.37, 0.76)	0.70 (0.46, 1.08)	0.62 (0.39, 0.98)	0.81 (0.52, 1.26)	0.69 (0.43, 1.11)	0.80 (0.47, 1.36)
Quartile 4	98/351	0.32 (0.23, 0.44)	0.53 (0.34, 0.83)	0.34 (0.22, 0.52)	0.57 (0.34, 0.96)	0.44 (0.28, 0.67)	0.61 (0.38, 0.97)
*p* for trend	474/948	<0.001	0.007	<0.001	0.010	<0.001	0.016
Per 1 higher score	474/948	0.79 (0.75, 0.84)	0.88 (0.80, 0.95)	0.80 (0.74, 0.87)	0.89 (0.81, 0.98)	0.85 (0.78, 0.91)	0.90 (0.83, 0.98)

GDQS, Global Diet Quality Score; MDS, Mediterranean Diet Score. ^1^ Adjusted for total energy intake during pregnancy sociodemographic characteristics (maternal age, work, education, residence, and parity), and maternal health-related factors in early pregnancy (passive smoking, anemia, medication use, and folate/iron supplements use).

## Data Availability

The data present in this study are available on request from the corresponding authors.

## Supplementary Materials

The following supporting information can be downloaded at: https://www.mdpi.com/article/10.3390/nu14173654/s1, Table S1: The constitution of congenital heart defects subtypes in the cases; Table S2: Daily nutrients intakes during pregnancy by Global Diet Quality Score category among cases and controls; Table S3: Daily nutrients intakes during pregnancy by Mediterranean Diet Score category among cases and controls; Figure S1: Subgroup analyses for the risk of total congenital heart defects associated with per 1 higher score of the Global Diet Quality Score; Figure S2: Subgroup analyses for the risk of congenital heart defects associated with per 1 higher score of the Mediterranean Diet Score.

## References

[1] Liu Y., Chen S., Zühlke L., Black G.C., Choy M.K., Li N., Keavney B.D. (2019). Global birth prevalence of congenital heart defects 1970–2017: Updated systematic review and meta-analysis of 260 studies. Int. J. Epidemiol..

[2] Zhao Q.M., Liu F., Wu L., Ma X.J., Niu C., Huang G.Y. (2019). Prevalence of Congenital Heart Disease at Live Birth in China. J. Pediatr..

[3] Zimmerman M.S., Smith A.G.C., Sable C.A., Echko M.M., Wilner L.B., Olsen H.E., Atalay H.T., Awasthi A., Bhutta Z.A., Boucher J.L. (2020). Global, regional, and national burden of congenital heart disease, 1990–2017: A systematic analysis for the Global Burden of Disease Study 2017. Lancet Child Adolesc. Health.

[4] Nie X., Liu X., Wang C., Wu Z., Sun Z., Su J., Yan R., Peng Y., Yang Y., Wang C. (2022). Assessment of evidence on reported non-genetic risk factors of congenital heart defects: The updated umbrella review. BMC Pregnancy Childbirth.

[5] Zhang T.N., Wu Q.J., Liu Y.S., Lv J.L., Sun H., Chang Q., Liu C.F., Zhao Y.H. (2021). Environmental Risk Factors and Congenital Heart Disease: An Umbrella Review of 165 Systematic Reviews and Meta-Analyses with More Than 120 Million Participants. Front. Cardiovasc. Med..

[6] Yang J., Kang Y., Chang Q., Zhang B., Liu X., Zeng L., Yan H., Dang S. (2022). Maternal Zinc, Copper, and Selenium Intakes during Pregnancy and Congenital Heart Defects. Nutrients.

[7] Zhang R., Guo L., Zhao D., Qu P., Dang S., Yan H. (2021). Maternal B-vitamin intake and B-vitamin supplementation during pregnancy in relation to neonatal congenital heart defects: A case-control study with propensity score matching. Eur. J. Clin. Nutr..

[8] Yang J., Kang Y., Cheng Y., Zeng L., Shen Y., Shi G., Liu Y., Qu P., Zhang R., Yan H. (2020). Iron intake and iron status during pregnancy and risk of congenital heart defects: A case-control study. Int. J. Cardiol..

[9] Qu Y., Lin S., Zhuang J., Bloom M.S., Smith M., Nie Z., Mai J., Ou Y., Wu Y., Gao X. (2020). First-Trimester Maternal Folic Acid Supplementation Reduced Risks of Severe and Most Congenital Heart Diseases in Offspring: A Large Case-Control Study. J. Am. Heart Assoc..

[10] Smedts H.P., Rakhshandehroo M., Verkleij-Hagoort A.C., de Vries J.H., Ottenkamp J., Steegers E.A., Steegers-Theunissen R.P. (2008). Maternal intake of fat, riboflavin and nicotinamide and the risk of having offspring with congenital heart defects. Eur. J. Nutr..

[11] Smedts H.P., de Vries J.H., Rakhshandehroo M., Wildhagen M.F., Verkleij-Hagoort A.C., Steegers E.A., Steegers-Theunissen R.P. (2009). High maternal vitamin E intake by diet or supplements is associated with congenital heart defects in the offspring. BJOG.

[12] Luo M., Wang T., Huang P., Zhang S., Song X., Sun M., Liu Y., Wei J., Shu J., Zhong T. (2022). Association and Interaction Effect of BHMT Gene Polymorphisms and Maternal Dietary Habits with Ventricular Septal Defect in Offspring. Nutrients.

[13] Luo M., Wang T., Huang P., Zhang S., Song X., Sun M., Liu Y., Wei J., Shu J., Zhong T. (2021). Association of maternal dietary intakes and CBS gene polymorphisms with congenital heart disease in offspring. Int. J. Cardiol..

[14] Zhang S., Liu X., Yang T., Wang T., Chen L., Qin J. (2022). Association of maternal dietary habits and ADIPOQ gene polymorphisms with the risk of congenital heart defects in offspring: A hospital-based case-control study. Eur. J. Clin. Nutr..

[15] Yang J., Kang Y., Cheng Y., Zeng L., Yan H., Dang S. (2019). Maternal Dietary Patterns during Pregnancy and Congenital Heart Defects: A Case-Control Study. Int. J. Environ. Res. Public Health..

[16] Sotres-Alvarez D., Siega-Riz A.M., Herring A.H., Carmichael S.L., Feldkamp M.L., Hobbs C.A., Olshan A.F. (2013). Maternal dietary patterns are associated with risk of neural tube and congenital heart defects. Am. J. Epidemiol..

[17] Obermann-Borst S.A., Vujkovic M., de Vries J.H., Wildhagen M.F., Looman C.W., de Jonge R., Steegers E.A., Steegers-Theunissen R.P. (2011). A maternal dietary pattern characterised by fish and seafood in association with the risk of congenital heart defects in the offspring. BJOG.

[18] Botto L.D., Krikov S., Carmichael S.L., Munger R.G., Shaw G.M., Feldkamp M.L. (2016). Lower rate of selected congenital heart defects with better maternal diet quality: A population-based study. Arch. Dis. Child. Fetal Neonatal Ed..

[19] Arimond M., Wiesmann D., Becquey E., Carriquiry A., Daniels M.C., Deitchler M., Fanou-Fogny N., Joseph M.L., Kennedy G., Martin-Prevel Y. (2010). Simple food group diversity indicators predict micronutrient adequacy of women’s diets in 5 diverse, resource-poor settings. J. Nutr..

[20] Madzorera I., Isanaka S., Wang M., Msamanga G.I., Urassa W., Hertzmark E., Duggan C., Fawzi W.W. (2020). Maternal dietary diversity and dietary quality scores in relation to adverse birth outcomes in Tanzanian women. Am. J. Clin. Nutr..

[21] Bromage S., Batis C., Bhupathiraju S.N., Fawzi W.W., Fung T.T., Li Y., Deitchler M., Angulo E., Birk N., Castellanos-Gutiérrez A. (2021). Development and Validation of a Novel Food-Based Global Diet Quality Score (GDQS). J. Nutr..

[22] Amati F., Hassounah S., Swaka A. (2019). The Impact of Mediterranean Dietary Patterns During Pregnancy on Maternal and Offspring Health. Nutrients.

[23] Qu Y., Deng X., Lin S., Han F., Chang H.H., Ou Y., Nie Z., Mai J., Wang X., Gao X. (2021). Using Innovative Machine Learning Methods to Screen and Identify Predictors of Congenital Heart Diseases. Front. Cardiovasc. Med..

[24] Liang Y., Li X., Hu X., Wen B., Wang L., Wang C. (2020). A predictive model of offspring congenital heart disease based on maternal risk factors during pregnancy: A hospital based case-control study in Nanchong City. Int. J. Med. Sci..

[25] Luo Y., Li Z., Guo H., Cao H., Song C., Guo X., Zhang Y. (2017). Predicting congenital heart defects: A comparison of three data mining methods. PLoS ONE.

[26] Li H., Luo M., Zheng J., Luo J., Zeng R., Feng N., Du Q., Fang J. (2017). An artificial neural network prediction model of congenital heart disease based on risk factors: A hospital-based case-control study. Medicine.

[27] Crozier S.R., Robinson S.M., Godfrey K.M., Cooper C., Inskip H.M. (2009). Women’s dietary patterns change little from before to during pregnancy. J. Nutr..

[28] Yang J., Cheng Y., Zeng L., Dang S., Yan H. (2021). Maternal dietary diversity during pregnancy and congenital heart defects: A case-control study. Eur. J. Clin. Nutr..

[29] Cheng Y., Yan H., Dibley M.J., Shen Y., Li Q., Zeng L. (2008). Validity and reproducibility of a semi-quantitative food frequency questionnaire for use among pregnant women in rural China. Asia Pac. J. Clin. Nutr..

[30] Yang J., Dang S., Cheng Y., Qiu H., Mi B., Jiang Y., Qu P., Zeng L., Wang Q., Li Q. (2017). Dietary intakes and dietary patterns among pregnant women in Northwest China. Public Health Nutr..

[31] Yang J., Cheng Y., Pei L., Jiang Y., Lei F., Zeng L., Wang Q., Li Q., Kang Y., Shen Y. (2017). Maternal iron intake during pregnancy and birth outcomes: A cross-sectional study in Northwest China. Br. J. Nutr..

[32] Institute of Nutrition and Food Safety, China Center for Disease Control (2005). China Food Composition Book 2.

[33] Institute of Nutrition and Food Safety, China Center for Disease Control (2009). China Food Composition Book 1.

[34] Mahmassani H.A., Switkowski K.M., Scott T.M., Johnson E.J., Rifas-Shiman S.L., Oken E., Jacques P.F. (2022). Maternal diet quality during pregnancy and child cognition and behavior in a US cohort. Am. J. Clin. Nutr..

[35] Mickey R.M., Greenland S. (1989). The impact of confounder selection criteria on effect estimation. Am. J. Epidemiol..

[36] Swets J.A. (1988). Measuring the accuracy of diagnostic systems. Science.

[37] Carmichael S.L., Yang W., Feldkamp M.L., Munger R.G., Siega-Riz A.M., Botto L.D., Shaw G. (2012). Reduced risks of neural tube defects and orofacial clefts with higher diet quality. Arch. Pediatr. Adolesc. Med..

[38] Feldkamp M.L., Krikov S., Botto L.D., Shaw G.M., Carmichael S.L. (2014). Better diet quality before pregnancy is associated with reduced risk of gastroschisis in Hispanic women. J. Nutr..

[39] Morales E., García-Serna A.M., Larqué E., Sánchez-Campillo M., Serrano-Munera A., Martinez-Graciá C., Santaella-Pascual M., Suárez-Martínez C., Vioque J., Noguera-Velasco J.A. (2022). Dietary Patterns in Pregnancy and Biomarkers of Oxidative Stress in Mothers and Offspring: The NELA Birth Cohort. Front. Nutr..

[40] Carmichael S.L., Ma C., Feldkamp M.L., Munger R.G., Olney R.S., Botto L.D., Shaw G.M., Correa A. (2012). Nutritional factors and hypospadias risks. Paediatr. Perinat. Epidemiol..

[41] Fisher S.A., Burggren W.W. (2007). Role of hypoxia in the evolution and development of the cardiovascular system. Antioxid. Redox Signal..

[42] Liu S., Joseph K.S., Lisonkova S., Rouleau J., Van den Hof M., Sauve R., Kramer M.S., Canadian Perinatal Surveillance System (Public Health Agency of Canada) (2013). Association between maternal chronic conditions and congenital heart defects: A population-based cohort study. Circulation.

[43] Bunin G.R., Gyllstrom M.E., Brown J.E., Kahn E.B., Kushi L.H. (2001). Recall of diet during a past pregnancy. Am. J. Epidemiol..

[44] Bosco J.L., Tseng M., Spector L.G., Olshan A.F., Bunin G.R. (2010). Reproducibility of reported nutrient intake and supplement use during a past pregnancy: A report from the Children’s Oncology Group. Paediatr. Perinat. Epidemiol..

[45] Zheng Z., Yang T., Chen L., Wang L., Zhang S., Wang T., Zhao L., Ye Z., Chen L., Qin J. (2018). Increased maternal Body Mass Index is associated with congenital heart defects: An updated meta-analysis of observational studies. Int. J. Cardiol..

