# Supplementary material for: Dietary Quality during Pregnancy and Congenital Heart Defects
Source: Nutrients. 2022 Sep 4;14(17):3654. doi: 10.3390/nu14173654 (PMC9460731; doi:10.3390/nu14173654)
Supplement: Supplementary file 1 [file nutrients-14-03654-s001.zip › nutrients-1853036-supplementary.pdf]

Table S1. The constitution of congenital heart defects subtypes in the cases.

Subtypes	Number	Proportion, %
Ventricular heart defects	222	46.84
Septal heart defects	218	45.99
Atrioventricular septal defects	87	18.35
Patent ductus arteriosus	74	15.61
Tetralogy of fallot	29	6.12
Pulmonary stenosis	17	3.59
Aortic valve stenosis	14	2.95
Transposition of the great arteries	7	1.48
Single ventricle	5	1.05
Others	2	0.42

A fetus with congenital heart defects may be diagnosed with two or more subtypes of congenital heart defects.

Table S2. Daily nutrients intakes during pregnancy by Global Diet Quality Score category among cases and controls [1].

	Case (N = 474)			Control (N = 948)		
	Quartile 1	Quartile 4	P	Quartile 1	Quartile 4	P
Total energy, kcal	1467.9 (1286.6, 1692.0)	2319.8 (2098.2, 2733.1)	< 0.001	1395.6 (1226.6, 1578.5)	2453.6 (2148.4, 2886.4)	< 0.001
Protein, g	31.5 (25.1, 41.0)	73.7 (64.4, 99.6)	< 0.001	33.0 (26.7, 42.9)	81.6 (65.9, 109.6)	< 0.001
Fat, g	19.3 (13.7, 30.0)	54.1 (40.8, 71.6)	< 0.001	24.0 (18.2, 34.7)	58.4 (46.0, 77.9)	< 0.001
Monounsaturated fatty acid, g	4.1 (2.6, 7.1)	13.1 (8.7, 19.4)	< 0.001	5.8 (4.3, 8.4)	13.1 (9.8, 17.8)	< 0.001
Saturated fatty acid, g	8.6 (5.8, 13.6)	21.2 (17.2, 27.5)	< 0.001	11.2 (8.7, 14.8)	23.6 (18.2, 33.7)	< 0.001
Carbohydrate, g	154.0 (123.7, 190.4)	263.0 (231.5, 317.1)	< 0.001	119.6 (98.1, 144.5)	283.6 (224.2, 362.1)	< 0.001
Iron, mg	13.8 (9.0, 17.5)	28.9 (23.3, 35.4)	< 0.001	11.3 (8.7, 14.8)	29.1 (24.1, 40.9)	< 0.001
Zinc, mg	3.1 (2.3, 4.3)	8.9 (6.8, 11.6)	< 0.001	3.6 (2.9, 4.8)	9.2 (7.4, 12.9)	< 0.001
Selenium, μg	16.0 (11.5, 22.9)	40.1 (32.2, 61.2)	< 0.001	18.2 (14.8, 24.3)	43.7 (33.7, 63.5)	< 0.001
Calcium, mg	302.8 (261.3, 423.2)	642.3 (545.5, 923.4)	< 0.001	303.1 (224.0, 389.9)	733.7 (577.8, 973.0)	< 0.001
Niacin, mg	7.5 (5.8, 9.6)	16.2 (11.2, 23.9)	< 0.001	7.2 (5.5, 9.7)	18.0 (14.4, 23.8)	< 0.001
Folate, μg	161.7 (147.1, 190.1)	280.7 (226.9, 350.1)	< 0.001	168.7 (153.1, 190.2)	276.5 (234.1, 374.6)	< 0.001
Vitamin C, mg	43.2 (30.7, 68.3)	122.2 (89.3, 180.0)	< 0.001	47.7 (37.1, 59.2)	136.9 (88.5, 218.0)	< 0.001

[1] Values are present as median (25th percentile, 75th percentile), and compared between groups by Mann–Whitney U test.

Table S3. Daily nutrients intakes during pregnancy by Mediterranean Diet Score category among cases and controls [1].

	Case (N = 474)			Control (N = 948)		
	Quartile 1	Quartile 4	*P*	Quartile 1	Quartile 4	*P*
Total energy, kcal	1418.3 (1243.6, 1622.7)	2274.0 (1934.5, 2623.5)	< 0.001	1361.9 (1206.5, 1535.5)	2445.8 (2078.9, 2866.4)	< 0.001
Protein, g	30.3 (23.6, 39.4)	69.7 (56.2, 88.1)	< 0.001	31.6 (25.4, 39.0)	80.3 (63.5, 104.4)	< 0.001
Fat, g	18.6 (13.6, 26.4)	51.2 (36.5, 67.6)	< 0.001	22.9 (17.5, 31.0)	57.5 (44.4, 79.4)	< 0.001
Monounsaturated fatty acid, g	3.6 (2.5, 6.1)	13.1 (8.1, 18.3)	< 0.001	5.3 (4.0, 7.4)	13.3 (9.7, 18.6)	< 0.001
Saturated fatty acid, g	8.3 (6.3, 11.4)	19.0 (15.5, 25.7)	< 0.001	10.4 (8.2, 14.5)	22.8 (17.7, 33.9)	< 0.001
Carbohydrate, g	138.3 (115.5, 174.5)	248.6 (214.3, 303.9)	< 0.001	116.6 (93.2, 142.1)	275.2 (212.3, 353.5)	< 0.001
Iron, mg	12.3 (7.6, 16.5)	25.8 (20.6, 33.4)	< 0.001	11.3 (8.6, 14.9)	28.5 (22.2, 37.9)	< 0.001
Zinc, mg	2.7 (2.1, 3.8)	8.4 (6.5, 10.7)	< 0.001	3.4 (2.8, 4.3)	9.1 (7.1, 12.5)	< 0.001
Selenium, μg	15.1 (11.3, 20.6)	36.1 (26.8, 49.7)	< 0.001	17.6 (13.8, 23.1)	43.2 (32.9, 60.1)	< 0.001
Calcium, mg	295.6 (246.8, 365.0)	633.0 (511.6, 813.3)	< 0.001	378.2 (312.0,465.8)	710.5 (562.3, 927.0)	< 0.001
Niacin, mg	7.3 (5.3, 9.3)	14.9 (10.3, 19.7)	< 0.001	7.1 (5.2, 9.1)	17.3 (14.0, 22.5)	< 0.001
Folate, μg	155.4 (144.3, 173.1)	274.9 (223.6, 326.4)	< 0.001	165.9 (151.9, 184.4)	270.0 (232.0, 360.8)	< 0.001
Vitamin C, mg	39.3 (28.2, 51.0)	118.0 (81.2, 183.4)	< 0.001	45.8 (36.8, 56.5)	119.7 (82.0, 200.7)	< 0.001

[1] Values are present as median (25th percentile, 75th percentile), and compared between groups by Mann–Whitney U test.

Subgroups	$N_{(cases)}$	$N_{(controls)}$	OR (95%CI)	*P* for interaction
**Maternal age**				0.759
<30 years	315	624	0.89 (0.85, 0.93)	
≥30 years	159	324	0.86 (0.80, 0.91)	
**Maternal work**				0.969
In employment	240	747	0.88 (0.84, 0.91)	
Without employment	234	201	0.88 (0.83, 0.93)	
**Maternal education**				0.009
Junior high school or below	195	183	0.83 (0.76, 0.90)	
Senior high shool or above	279	765	0.90 (0.86, 0.94)	
**Residence**				0.008
Urban	313	679	0.94 (0.89, 0.98)	
Rural	161	269	0.87 (0.83, 0.91)	
**Parity**				0.066
0	274	761	0.89 (0.85, 0.93)	
≥1	200	187	0.87 (0.82, 0.93)	
**Passive smoking**				0.903
No	315	860	0.88 (0.85, 0.92)	
Yes	159	88	0.86 (0.80, 0.93)	
**Anemia**				0.719
No	394	845	0.87 (0.84, 0.91)	
Yes	80	103	0.90 (0.86, 0.95)	
**Medication use**				0.245
No	277	660	0.89 (0.86, 0.93)	
Yes	197	288	0.86 (0.81, 0.91)	
**Folate/iron supplements use**				0.702
Yes	363	846	0.87 (0.84, 0.91)	
No	111	102	0.91 (0.82, 0.98)	
**Overall**	474	948	0.88 (0.85, 0.91)	

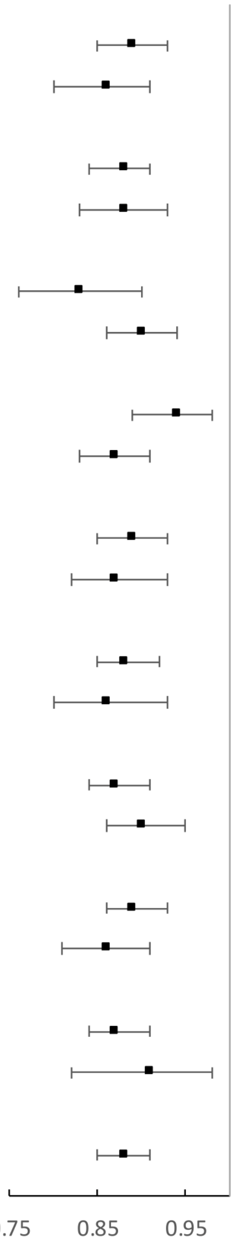

Figure S1. Subgroup analyses for the risk of total congenital heart defects associated with per 1 higher score of the Global Diet Quality Score.

Subgroups	$N_{(cases)}$	$N_{(controls)}$	OR (95%CI)	P for interaction
**Maternal age**				0.845
< 30 years	315	624	0.87 (0.78, 0.97)	
≥ 30 years	159	324	0.86 (0.74, 0.99)	
**Maternal work**				0.581
In employment	240	747	0.87 (0.78, 0.97)	
Without employment	234	201	0.88 (0.76, 1.02)	
**Maternal education**				0.130
Junior high school or below	195	183	0.84 (0.69, 1.01)	
Senior high shool or above	279	765	0.90 (0.81, 0.99)	
**Residence**				0.004
Urban	313	679	0.93 (0.81, 1.06)	
Rural	161	269	0.84 (0.74, 0.95)	
**Parity**				0.082
0	274	761	0.90 (0.81, 0.99)	
≥1	200	187	0.84 (0.72, 0.98)	
**Passive smoking**				0.827
No	315	860	0.90 (0.81, 0.99)	
Yes	159	88	0.78 (0.65, 0.95)	
**Anemia**				0.523
No	394	845	0.83 (0.77, 0.89)	
Yes	80	103	0.86 (0.72, 1.04)	
**Medication use**				0.238
No	277	660	0.85 (0.77, 0.94)	
Yes	197	288	0.92 (0.80, 1.06)	
**Folate/iron supplements use**				0.250
Yes	363	846	0.87 (0.79, 0.96)	
No	111	102	0.87 (0.73, 1.04)	
**Overall**	474	948	0.88 (0.80, 0.95)	

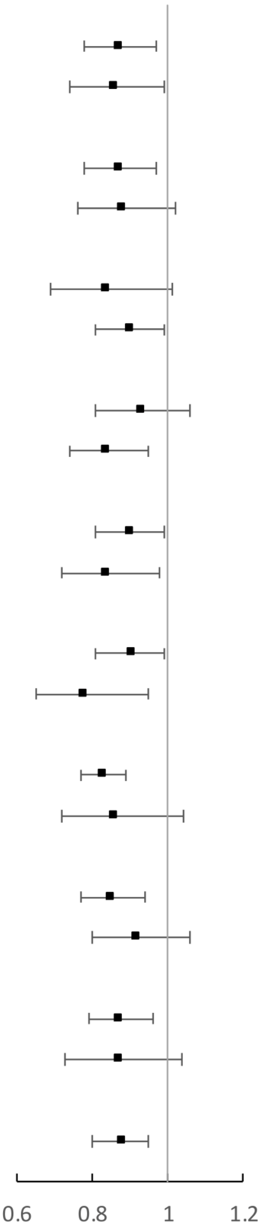

Figure S2. Subgroup analyses for the risk of total congenital heart defects associated with per 1 higher score of the Mediterranean Diet Score.
